# Dietary Behavior of *Drosophila melanogaster* Fed with Genetically-Modified Corn or Roundup^®^

**DOI:** 10.3390/jox11040014

**Published:** 2021-12-17

**Authors:** Raquel Elias, Becky Talyn, Erik Melchiorre

**Affiliations:** 1 Department of Biology, California State University, San Bernardino, CA 92407, USA; elias1raquel@gmail.com; 2 College of Natural Sciences, California State University, San Bernardino, CA 92407, USA; 3 Department of Geology, California State University, San Bernardino, CA 92407, USA; emelch@csusb.edu

**Keywords:** *Drosophila melanogaster*, glyphosate, organic, Roundup^®^, GMO, diet, behavior, pelargonic acid, POEA

## Abstract

With the rise in concern about GMOs and pesticides on human health, we have utilized *Drosophila melanogaster* as a model organism for understanding the effects of Roundup-Ready^®^ GMO diets on health. We recorded dietary behavior during and after exposure to a medium containing GMO or non-GMO corn, Roundup^®^ in organic corn medium, and sucrose with or without one of the two Roundup^®^ formulations. No differences in behavior were observed when *Drosophila* were exposed to a medium containing Roundup-Ready^®^ GMO or non-GMO corn. *Drosophila* can detect and refrain from eating sucrose containing one Roundup^®^ formulation, Ready-to-Use, which contains pelargonic acid in addition to glyphosate as an active ingredient. *Drosophila* exhibited dose-dependent increased consumption of sucrose alone after exposure to a medium containing either Roundup^®^ formulation. This may indicate that flies eating a medium with Roundup^®^ eat less and were thus hungrier when then given sucrose solution; that a medium with Roundup^®^ is more difficult to digest; or that a medium with Roundup^®^ is less nutritious, as would be the case if nutritionally important microbes grew on control medium, but not one containing Roundup^®^.

## 1. Introduction

With the rise of modern agriculture, the use of genetically modified foods and pesticides, including herbicides, has become increasingly prevalent worldwide. In an effort to minimize potential adverse health and environmental effects, the United States Department of Agriculture [1] developed Certified Organic standards, such as the restriction of artificial pesticides and herbicides, chemical fertilizers, irradiation, and genetically modified organisms (GMOs). While the principles of organic agriculture give it a perception of being healthier and environmentally safer than conventionally grown crops, its relevance to human health is still inconclusive [2]. However, growing evidence suggests that organic foods are more nutrient-dense than industrially grown, non-organic food, as well as being environmentally friendlier [3,4,5,6,7,8].

GMOs are strains of crops which contain DNA, usually from other species, which has been added so the crop will exhibit a desired characteristic not naturally occurring in that plant type. A common GMO trait is to be resistant against a particular herbicide, usually glyphosate-based herbicides (GBH) like Roundup^®^. As the DNA is inserted into the plant genome (genetically-modified), it is possible for DNA in the plant’s own genome to be altered unintentionally. Studies have shown unintentional consequences with GMOs, such as decrease in nutrients [3,5,8], no improvement in crop yield [9,10], increase in herbicide usage due to glyphosate resistance in crops such as soybeans [3,11], reduction in platelet aggregation [12], biochemical changes in rats [13,14], as well as insertions, deletions, and rearrangements in the DNA [15], and modifications in regulation of protein expression [14].

Although many agricultural toxins are sold, the herbicide glyphosate, the active ingredient in Roundup^®^, is a widely utilized post-emergence herbicide in landscaped areas [16]. It is non-selective, post-emergent, and directly obstructs the shikimate metabolic pathway necessary for plant development [17], but it can be slow-acting. Glyphosate has been branded as safe for human exposure, since animals do not possess the shikimate pathway; however, experimental results suggest that glyphosate can disrupt other developmental and metabolic processes in animals and humans. Glyphosate may disrupt reproduction [18,19,20,21,22,23,24], endocrine systems [20], feeding behavior [18,21], microbiomes [25,26,27], hepatorenal systems [26,28,29], nervous systems [30,31], mental health [25]; can cause indirect DNA damage [32]; can pass through food and feed as well as through the placenta [33,34]; can cause reversible encephalopathy at large doses [35] and alter morphophysiology [36]. Glyphosate residue concentrations ranging from 100 s to 1000 s ppb have been found in common foods [37], while rats were shown to have severe organ damage at just 0.05 ppb [29]. Given these alterations to multiple systems, it is important to further study the effects of glyphosate, and its commercial formulations, on non-target organisms.

Commercial GBH formulations especially harm animals because these herbicides expose them to adjuvants and secondary herbicides in conjunction with glyphosate [6,38,39,40]. Commercial formulations of Roundup^®^ include adjuvants such as the surfactant POEA (polyethoxylated tallowamine), which facilitates herbicide uptake in plants, and pelargonic acid or diquat, which are secondary herbicides that cause rapid breakdown of weeds at initial application [16,41]. Although effective and often faster-acting, these formulations, which are proprietary and complete ingredients lists, therefore confidential, have enhanced toxicity compared to glyphosate alone, as has already been shown in non-target organisms, including rodents [23] and aquatic organisms [6,42,43].

Field studies provide evidence of glyphosate’s toxicity to humans. Agricultural workers exposed to glyphosate tend to have glyphosate absorbed into their bodies and suffer renal tissue damage [22,28,37,44]; women in the agricultural San Juaquin Valley of California, exposed to high quantities of pesticides, experienced adverse birth outcomes [22] and shorter gestational periods [45]. People exposed to high levels of pesticides, for example, glyphosate, were more likely to develop Non-Hodgkin Lymphoma [46,47]; pesticide exposure was associated with higher risk of autism [32]; and chronically ill people had higher levels of glyphosate detected in their urine than healthy people [48].

Humans can reduce their exposure to glyphosate and other pesticides [5] and their pesticide load [37,44] by choosing to eat organic foods. Switching to an organic diet reduces urinary glyphosate levels after only three days, in both adults and children [49]. Other animals may also reduce their pesticide exposure by reducing intake of pesticide-laden food, if they are able to detect and avoid pesticides in food. Research already suggests that *Drosophila*, like humans, have the ability to discriminate against bitter and salty compounds [50]. Avoidance of pesticides by *Drosophila* could result from evolutionary selection, since at high concentrations, GBH increase mortality and decrease reproduction and ovary size [21,24,51], and GBH have been in use commercially since 1974, at least 800 generations for *Drosophila*. To further explore sub-lethal toxic effects of GMOs and GBH, and the ability of animals to detect and avoid food containing them, *Drosophila melanogaster* were exposed to glyphosate-tolerant GMO corn with low concentrations of pesticide residue, or to two formulations of Roundup^®^, to determine their effects on food intake and dietary behavior.

### Specific Hypotheses

**Hypothesis** **1** **(H1).** *Drosophila avoid medium containing Roundup-Ready^®^ GMO corn.*

**Hypothesis** **1a** **(H1a).** *Avoidance of Roundup-Ready® corn occurs whether or not the corn was sprayed with Roundup, because the genetic modification itself makes the corn less palatable.*

**Hypothesis** **1b** **(H1b).** *Avoidance of Roundup-Ready^®^ corn is maximized when the corn was treated with Roundup, because the herbicide is also avoided*.

**Hypothesis** **2** **(H2).** *Drosophila avoid medium containing GBH, even at sub-lethal concentrations*.

**Hypothesis** **3** **(H3).** *Drosophila exposed to a medium containing GBH eat less, become hungry, and therefore eat more when later provided with non-GBH food.*

## 2. Experimental Section

### 2.1. Fly Maintenance

*Drosophila melanogaster* fruit flies of the Canton-S strain were obtained from Dr. Erik Johnson at Wake Forest University, North Carolina. Stocks were maintained on corn-based medium in a 25 °C lab, under a 12 h light: 12 h dark cycle, in 250 mL bottles with ~50 mL medium. Medium was cooked in 300 mL batches consisting of 16.30 g Bob’s Redmill organic cornmeal, 3.34 g non-GMO nutritional yeast, 3.07 g wild-harvested agar, 360 mL water, 20 mL Wholesome organic molasses, and 0.9 mL propionic acid (added after other ingredients were cooked together to inhibit mold growth).

All adult flies used in feeding assays were collected and sexed four to six hours post eclosion, using CO_2_ anesthesia. After seven days, flies were starved for fifteen to sixteen hours in a clean vial containing two Kimwipes and two milliliters of water to prevent desiccation and were then immediately lightly anesthetized and transferred to a feeding assay.

### 2.2. Assay 1: Food Preference—Medium with GMO Corn

Flies in some experimental treatments were exposed to a medium containing one of three types of corn for their whole lifecycle. This corn was used in “blind treatments” (experimenter did not know which treatment was which until after the experiments were completed) and was either (A) corn genetically modified to be Roundup-Ready^®^ (also called glyphosate-resistant or herbicide-tolerant) and sprayed with Roundup^®^ Weathermax twice during growth, (B) Roundup-Ready^®^ GMO corn not sprayed with Roundup^®^ during growth, or (C) corn not genetically modified, but otherwise genetically the same (isogenic, according to provider Pioneer) and not sprayed with Roundup^®^ during growth. All three strains of corn were grown during the same growing season in adjacent fields in Iowa, USA. Aside from strain and Roundup spray, all three types of corn were grown in the same way [51]. Adult flies from lab stocks were introduced to 250 mL bottles containing medium made with one of these types of corn or a negative control, commercially available organic corn. Mixed sex groups were housed in these bottles and allowed to lay eggs. Adults used in these experiments developed from those eggs.

Newly eclosed adult flies were collected in groups of 20 males or females from a single treatment (A, B, or C corn or organic control; “exposure treatment”) and transferred to a vial with the same treatment they were exposed to as larvae. After seven days plus the starvation period, the 20 female or male flies were introduced into the bottom of a T-maze, designed based on those used in several studies [52,53,54] and tapped down until all were successfully introduced into the maze; then it was closed off with a cotton ball. Flies were allowed fifteen minutes to choose between their exposure treatment or a new treatment: organic if their exposure treatment was A, B, or C; and organic, A, B, or C if they were reared on organic. This approach, using multiple treatments for rearing, as well as exposure, prevented us from misinterpreting a preference for a familiar medium with that for a particular medium. In addition, this method was used to differentiate between choosing what they were used to and avoiding GMO medium. At the end of the fifteen minutes, each fly on each side of the T-maze was recorded or declared “undecided.” Undecided flies had not chosen a side and could not be seen in either side of the maze. A total of 20 trials for each of the seven treatments, and a maximum of 20 flies per trial, were used on a weekly basis for a total of 2874 flies.

We calculated a dispersal index and a preference index for each trial, using formulae from previous studies [55,56]:

$$Dispersal\;Index=\frac{1-N_{undecided}}{N_{introduced}}$$

$$Preference\;Index=\frac{N_{organic}-N_{conventional}}{N_{organic}+N_{conventional}}$$

in which $N_{introdced\;}$ represents the number of flies that were introduced into the T-maze at the beginning of the trial, $N_{undecided}$ indicates the number of flies that were not in either vial of the T-maze but rather remained in the base, $N_{Organic}$ represents the number of flies in the T-maze observed in the vial containing organic corn medium, and $N_{conventional}$ the number of flies observed in the vial containing either A, B, or C corn medium. These data were analyzed using JMP statistical software using Wilks λ multivariate ANOVA to assess the effects of sex and diet, and an F test to evaluate dispersal index and preference index separately. Since two separate analyses were completed, one for flies reared on organic medium and one for flies reared on other treatments, we applied a Bonferroni-type correction and significance of *p* < 0.025.

### 2.3. Assay 2: Food Preference—Sucrose with Roundup

Using the method from Toshima et al.’s two choice preference test [54], sucrose treatments were made with 20 g organic sugar and 100 mL water infused with red or blue dye for distinguishing between treatments. Sucrose solutions were left organic (negative control) or supplemented with 10 g/L glyphosate acid equivalent from one of two glyphosate-based Roundup^®^ formulations: Super Concentrate with the adjuvant/surfactant POEA, or Ready to Use with the secondary herbicide Pelargonic acid [24] (see Table 1). We used 10 g/L for this experiment, since, in our previous study [51], exposure to this concentration of Roundup^®^ in medium resulted in 100% mortality. Glyphosate-based Roundup^®^ formulations, rather than glyphosate alone, were used, since they are more environmentally relevant in both homeowner and agricultural applications.

For each trial, 10 newly eclosed female or male flies were collected from organic medium using CO_2_ anesthesia. All flies were transferred to organic medium for seven days. On the seventh day, flies were starved for 15–16 h, anesthetized again with CO_2_, and transferred to a 10 mL beaker containing two microcentrifuge tube caps, with one red and one blue 20% sucrose treatment. When neither sucrose treatment contained Roundup and differed only in color, flies exhibited no preference for red versus blue food dye (*t* = 0.8401, *p* = NS). Nonetheless, each sucrose treatment was presented with red in some trials and blue in others. One microcentrifuge cap always contained organic sucrose with red or blue dye, while the other contained 10 g/L of glyphosate from one of the two Roundup^®^ formulations with the other color dye. One hour was given for flies to consume sucrose, after which the abdomen of each fly was examined under a dissecting microscope to determine the color (red, blue, or purple/mixture) of sucrose consumed. Food remains in the *Drosophila* digestive system for about 1 hour [57], so examining abdominal color at this time should reflect most of the sucrose eaten. If the color of the abdomen could not be distinguished through the abdominal wall, the treatment consumed was determined by pressing on the abdomen to squeeze out a drop of the digestive fluid. A total of 300 flies were used in 53 trials, for an average of 6 flies per trial, due to pre-experimental loss of flies. We did not use quantitative methods for this experiment because the physical properties of the Roundup^®^ formulations prevented the mixtures from staying in graduated capillary tubes. Since this experiment focused on qualitative (color of abdomen) rather than quantitative measures (how much sucrose was consumed), it was reasonable to use the centrifuge caps instead.

A matched pairs *t*-test was performed to compare response to blue or red sucrose. Four matched pairs *t*-tests by sex and formulation were also performed to compare consumption of organic vs. glyphosate-containing sucrose for male and female flies exposed to each of the two Roundup formulations, using a Bonferroni-corrected *p*-value of 0.0125 to define significance.

### 2.4. Assay 3: Sucrose Consumption after Roundup Exposure

Glyphosate treatments were made with the same organic corn medium used to maintain stocks, but with the addition of commercially available Roundup^®^ Super Concentrate or Roundup^®^ Ready to Use, added following the propionic acid. Concentrations of glyphosate in Roundup^®^ used were 0 g/L (negative control), 0.5 g/L, 1.0 g/L, and 2.0 g glyphosate acid equivalent/L medium, all considerably less than the LC_50_ previously reported, even for seven days of exposure [51].

In this experiment, we wanted to quantify how much sucrose was being consumed, unlike the previous assay. Using modifications of methods from Diegelmann et al.’s Café Assay [58], each trial started with collection of 30 newly eclosed female flies from lab stocks. Flies were transferred to a randomly selected treatment for seven days. After 15–16 h of starvation, flies were transferred to a clean vial without medium and covered with a plug in which we inserted a microcapillary tube filled with 5 µL of 20% organic sucrose solution infused with red or blue dye to facilitate measurement. After one hour, the total volume of sucrose consumed was recorded. An empty vial without flies, but with a microcapillary tube containing 5 µL of sucrose solution, served as a humidity control to determine the evaporation rate during the same hour of experimentation. The average sucrose consumption for each fly was calculated by determining the amount of sucrose consumed, rather than evaporated, using this formula

$$Sucrose\;Consumption=\frac{Volume\;change\;in\;vial\;i\;\left(with\;flies\right)-Volume\;change\;in\;vial\;0\;\left(without\;flies\right)}{Number\;of\;flies\;in\;vial\;i}$$

Each of the seven treatments was repeated ten times for a total of 1814 flies and an average of 26 flies per trial.

Using JMP statistical software, a linear regression was calculated for average sucrose consumption per fly at increasing glyphosate concentrations. Regression coefficients were calculated separately for each of the two Roundup^®^ formulations, using *p_critical_* < 0.025.

## 3. Results

### 3.1. Assay 1: Food Preference—Medium with GMO Corn

**Figure 1:**
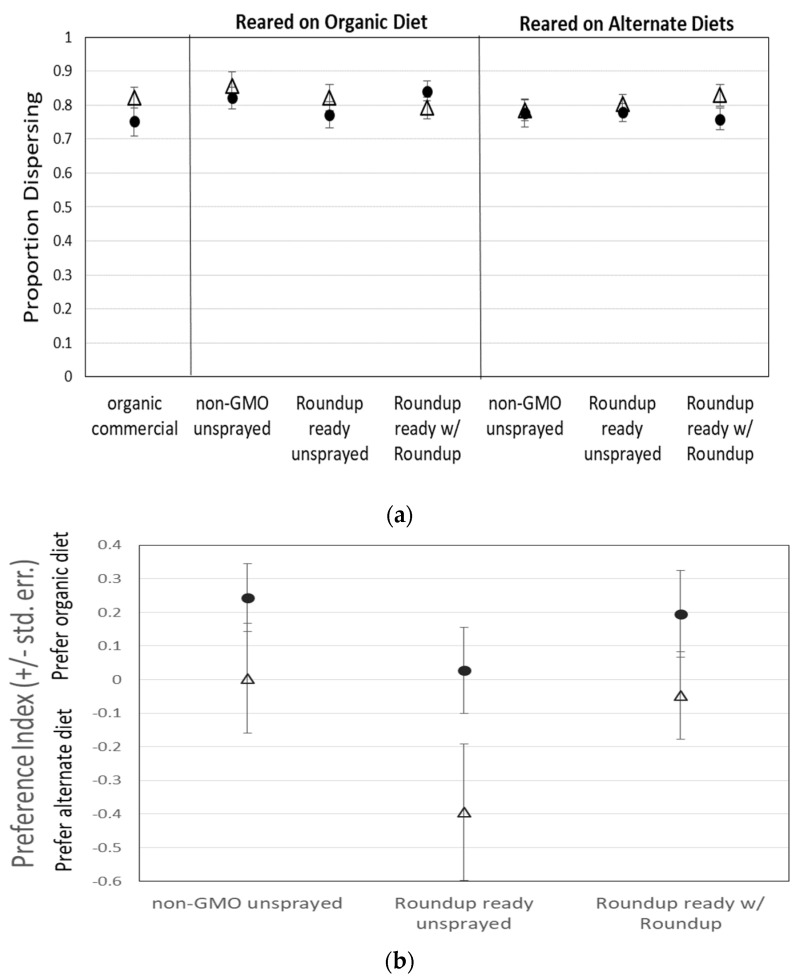

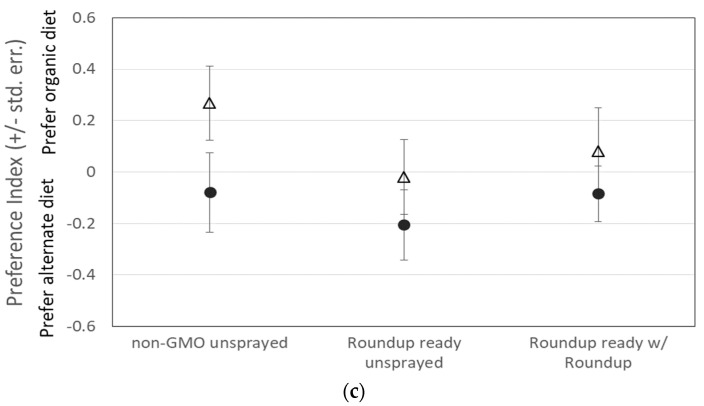
(**a**) The dispersal of 2874 flies for all treatments was not significantly different. Flies dispersed relatively consistently throughout all treatments. Preference for organic (+) or alternate (−) diet when flies were (**b**) reared on organic corn or (**c**) reared on the experimental treatment medium did not significantly differ, except that males were more likely than females to prefer the familiar diet, especially when reared on organic corn medium. λ = males, △ = females.

*Drosophila* feeding behavior was not influenced by the type of corn in their diet during the larval and early adult period, nor by the type of corn in the test medium. The dispersal index did not differ between any treatments, indicating that both male and female flies dispersed into the T-maze with the same likelihood, regardless of the type of corn they were fed during growth or exposed to within the T-maze (Figure 1a; Table 1). Therefore, any differences in preference index should result from a difference in attraction to or avoidance of a particular food source, not a difference in overall activity level, motivation, or attraction to food in general.

However, there was also no significant difference in preference index between any diet treatments (A, B, C, or organic) for either male or female flies, except that when reared on the organic diet, males were more likely than females to be found on the organic side of the T-maze, regardless of which medium was presented on the other side (Figure 1b; Table 1). There was also a non-significant tendency for males to be more likely than females to be found on the medium on which they were reared when reared on a diet containing non-organic corn (Figure 1c). It is also important to note that our GMO corn that was sprayed with Roundup^®^ was tested for residual glyphosate and AMPA (the primary metabolite of glyphosate) and was found to contain a combined total of only 0.4 µg/L.

### 3.2. Assay 2: Consumption of Sucrose with Roundup

**Figure 2:**
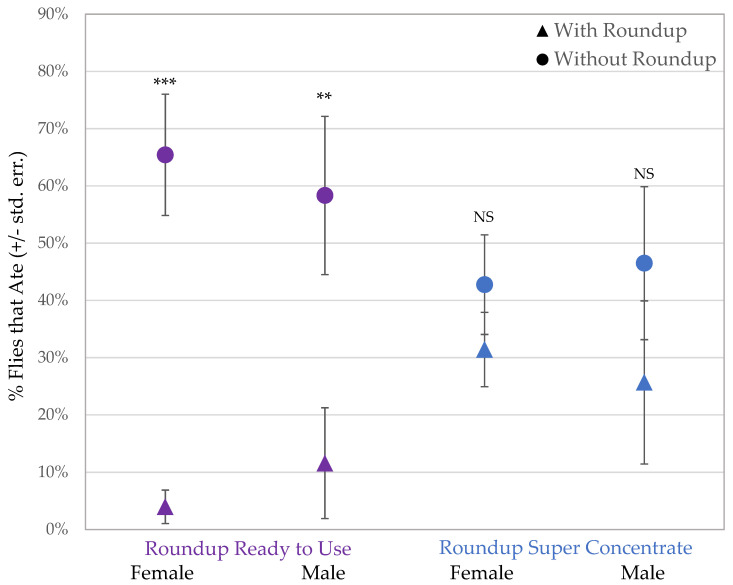
Female and male flies consumed significantly more organic sucrose in a 2-choice test when given the alternative option of sucrose with Roundup^®^ Ready to Use (purple/left; *** *p* < 0.001, ** *p* < 0.01). There was no difference in sucrose consumption during exposure to Roundup^®^ Super Concentrate sucrose or control sucrose (blue/right, NS *p* > 0.05).

The sucrose consumption assay presented a choice between organic sucrose and sucrose containing Roundup^®^ with either pelargonic acid or POEA to 94 males and 206 females. After a total of 300 flies tested, 91 flies did not consume either treatment and remained uncolored after the 1-h assay. Those flies that did consume sucrose preferred organic sucrose when given the option of sucrose mixed with Roundup^®^ Ready to Use, containing glyphosate and pelargonic acid (Figure 2; males: *t* = 2.255, *p* = 0.0027; females: *t* = 4.789, *p* = 0.0003). Both male and female flies showed no preference between organic sucrose and sucrose with Roundup^®^ Super Concentrate, which contains glyphosate and POEA (Figure 2; males: *t* = 0.8805, *p* = NS; females: *t* = 0.78688, *p* = NS).

### 3.3. Assay 3: Sucrose Consumption after Roundup Exposure

**Figure 3:**
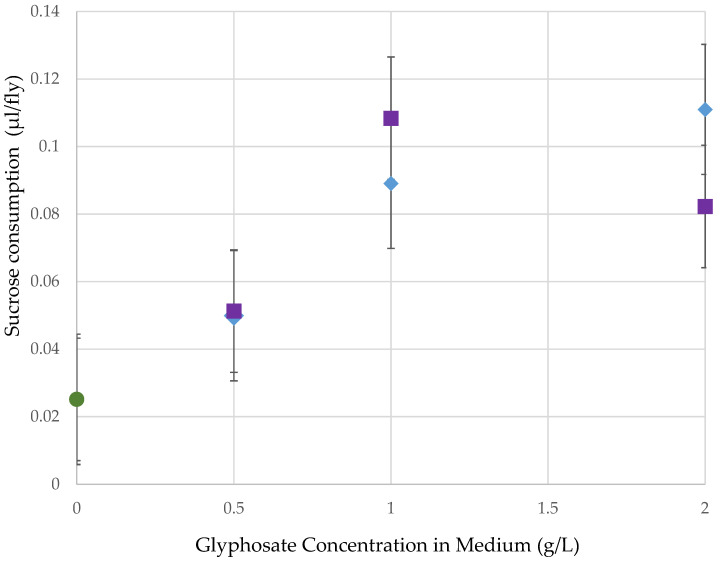
Of the total 1814 flies, there was an increase in organic sucrose consumption as the concentration of glyphosate in the pre-treatment medium increased (*p* = 0.001). There was no difference between the two formulations. ◯ = organic control, ☐ = Roundup Ready to Use (glyphosate + pelargonic acid), ◇ = Roundup Super Concentrate (glyphosate with POEA).

Female flies raised as adults on medium with high concentrations of glyphosate in Roundup later consumed more organic sucrose, resulting in a positive regression between glyphosate concentration in adult medium and sucrose consumption after 15–16 h of starvation (Figure 3; R^2^ = 17.8%, *p* = 0.001). No difference occurred between formulations (F = 0.0767, *p* = NS), so this factor was not considered in the overall regression analysis.

## 4. Discussion

While other studies show that behavior of model organisms can be altered by exposure to Roundup^®^ and other glyphosate-based herbicides [25,42], genetically modified Roundup-Ready^®^ corn did not influence dietary behavior in *Drosophila melanogaster*. In the first experiment (medium with GMO corn), the dispersal of flies was about the same for each treatment and trial, therefore T-maze preferences should not be attributed to general activity level, feeding motivation, or hunger. The 0.4 µg/L of glyphosate and AMPA detected in the medium made with GMO and Roundup^®^ sprayed corn was negligible compared to the concentrations we were testing and compared to the lowest concentration used in our previous study (0.1 g/L), which had no effect on fly mortality and no other obvious effects [51]. Therefore, if there had been a significant difference in corn source preferences, it would unlikely have been attributable to Roundup^®^ and its active ingredient, glyphosate, but rather to GMO corn. However, no variation among treatments was observed, though there was a trend for males to more often be observed on the diet to which they had been previously exposed. This might indicate the ability to distinguish among the treatments [59], even if there is no clear preference for a particular type of corn, though it was a weak and inconclusive effect. The sex difference could partly be due to differences in energy expenditure and nutrient demand of male and female *Drosophila*. Female fruit flies tend to need more nutrients for the demands of reproduction than males and have been shown to be more sensitive to dietary restrictions [60], causing females to be less selective of food sources.

Although the presence of genetically modified corn failed to alter dietary preference, there is a possibility of gut microbiome alterations, as this has been seen in other species from ingestion of GMO corn [61]. Studies suggest that GMO foods may affect an organism’s morphology, protein expression, gastrointestinal tract histology, and the nutrition gained from food crops [8,61,62]. Given this information, it is important to explore other organ systems GMOs may affect, since there is evidence of potential dangers in other types of organisms [61]. While the Roundup^®^ Ready gene itself is unlikely to be the cause of these changes, there is a variety of mechanisms associated with the process of DNA insertion that might account for them (reviewed in [51]).

When flies were given the choice of organic sucrose or sucrose with Roundup^®^ Ready to Use, containing 10 g/L of glyphosate and pelargonic acid, more flies consumed organic sucrose. However, when flies had the choice between organic sucrose and sucrose with Roundup^®^ Super Concentrate, containing 10 g/L of glyphosate and POEA, there was no difference. This supports previous evidence [39,63,64] that the formulation of Roundup^®^ and the presence of ingredients other than glyphosate change its bioactive properties and can modify the effects of exposure or the ability to detect its presence. However, the adjuvant POEA is generally thought to be one of the important ingredients in toxicity [39,63,64], while pelargonic acid has not been well studied. These data suggest that *Drosophila* may not detect or respond to glyphosate or POEA, but they are able to detect and avoid pelargonic acid. Since many different Roundup^®^ formulations are sprayed in private and public spaces, on crops for human consumption, and end up in the environment, it is important that each of these formulations be evaluated to assess potential safety concerns.

Flies given medium as adults that contained various concentrations of glyphosate (0 g/L, 0.5 g/L, 1.0 g/L, or 2.0 g/L) in Roundup^®^ Ready to Use or Roundup^®^ Super-Concentrate later consumed more organic sucrose after higher concentrations of glyphosate exposure. In this case, glyphosate itself is likely to be the factor responsible for altering sucrose consumption, since flies exhibited very similar dose response to both Roundup^®^ formulations. This may reflect that flies were choosing not to consume medium containing glyphosate and so were more starved before being allowed to consume the organic sucrose. This is consistent with the observation of Aguiar, et al. [21] that concentration of glyphosate exposure correlates with female body mass, possibly because of reduced food consumption. Another possibility is that nutritionally important microbes that reside on the medium became depleted or disrupted by the introduction of Roundup^®^, which is known to have anti-microbial properties [27], leading to the flies being deprived of an important food source, and hence, being hungrier [65]. This also might account for some of the reduction in ovary size and reproduction seen by Muller et al. [24], since dietary yeast increase fecundity [66].

### Future Directions

Further studies are needed to distinguish between possible reasons why flies are choosing one food source over another. To confirm if flies choose to not consume Roundup^®^, proboscis extension behavior should be monitored, or the FLIC system used to distinguish between tasting and feeding. An evaluation of 16S and 18S ribosomal RNA from microbial colonies on the surface of the medium and in *Drosophila* digestive systems might elucidate the role of glyphosate-induced changes in microbial communities. It would also be useful to investigate the ingredients in Roundup^®^ formulations independently and in combination to understand more precisely which components alter fly feeding behavior.

## 5. Conclusions

Our studies reinforce the need for reevaluation of commercial and agricultural glyphosate formulations and herbicide-tolerant GMOs, including at sub-lethal levels. Although we did not see a preference between Roundup-Ready^®^ GMO corn and non-GMO corn, in a feeding choice experiment, one of the two Roundup^®^ herbicide formulations did result in a preference for herbicide-free sucrose. It is important to note that GMO corn alone did not result in a difference in food preference behavior, but added Roundup^®^ altered *Drosophila*’s food preference at sub-lethal doses, depending on the other ingredients in the formulations, possibly POEA or pelargonic acid. Specifically, fruit flies were able to detect the Ready to Use formulation with pelargonic acid and preferred organic sucrose to it. Longer-term exposure to the two Roundup^®^ formulations, administered in medium, resulted in those flies exposed to higher glyphosate concentrations being partially starved and therefore consuming more organic sucrose. Since this is a gain of function behavioral response, it is not caused by general toxicity impairing locomotion, activity level, or feeding ability.

This study does not rule out the potential toxic effects of GMOs, since other studies have shown, for example, possible toxicity of GMO corn [61] and soybeans. Rather, this study shows that *Drosophila melanogaster* may be able to distinguish between corn sources (males more sensitively) and sucrose with vs. without Roundup. It is likely that glyphosate and GBH play a bigger role in feeding behavior than GMOs and may have other sub-lethal affects. Additional work is critical to determine the safety of GMOs and herbicides, especially glyphosate, since it is so widely used. With the increasing awareness and number of studies about organic foods, people have become concerned with their health and safety when consuming non-organic foods. Although there is no conclusive evidence that GMOs and herbicides are safe for human health, our results corroborate many recent studies that provide evidence that they are not.

## Figures and Tables

**Table 1:** Statistical results from Wilks’ λ, multivariate ANOVA, and F-test for Dispersal Index and Preference Index.

		Multivariate		Dispersal Index		Preference Index	
		F	*p*	F	*p*	F	*p*
Flies reared on organic diet	Overall	1.5732	NS	0.9438	NS	2.2213	NS
	Diet	1.9016	NS	0.8211	NS	3.0717	NS
	Sex	5.1857	0.0078	1.0682	NS	8.8508	0.0039
	Interaction	0.6494	NS	1.1123	NS	0.1697	NS
Flies reared on treatment diet	Overall	1.1721	NS	0.6488	NS	1.731	NS
	Diet	1.0932	NS	0.0594	NS	2.2143	NS
	Sex	2.3102	NS	3.3639	NS	0.8941	NS
	Interaction	0.9749	NS	0.4277	NS	1.6823	NS

## Data Availability

The data upon which this article is based is found within the article in the figures and tables. More detailed versions of these data are available upon request from the corresponding author.
